# Dynamics of Maize Vegetative Growth and Drought Adaptability Using Image-Based Phenotyping Under Controlled Conditions

**DOI:** 10.3389/fpls.2021.652116

**Published:** 2021-05-11

**Authors:** Dejan Dodig, Sofija Božinović, Ana Nikolić, Miroslav Zorić, Jelena Vančetović, Dragana Ignjatović-Micić, Nenad Delić, Kathleen Weigelt-Fischer, Thomas Altmann, Astrid Junker

**Affiliations:** ^1^Department for Research and Development, Maize Research Institute Zemun Polje, Belgrade-Zemun, Serbia; ^2^Department for Maize, Institute for Field and Vegetable Crops, Novi Sad, Serbia; ^3^Department of Molecular Genetics, Leibniz Institute of Plant Genetics and Crop Plant Research, Gatersleben, Germany

**Keywords:** maize, image-derived traits, growth dynamics, temporal profiles, drought capabilities

## Abstract

Changes in climate are likely to have a negative impact on water availability and soil fertility in many maize-growing agricultural areas. The development of high-throughput phenotyping platforms provides a new prospect for dissecting the dynamic complex plant traits such as abiotic stress tolerance into simple components. The growth phenotypes of 20 maize (*Zea mays* L.) inbred lines were monitored in a non-invasive way under control, nitrogen, and water limitation as well as under combined nitrogen and water stress using an automated phenotyping system in greenhouse conditions. Thirteen biomass-related and morphophysiological traits were extracted from RGB images acquired at 33 time points covering developmental stages from leaf count 5 at the first imaging date to leaf count 10–13 at the final harvest. For these traits, genetic differences were identified and dynamic developmental trends during different maize growth stages were analyzed. The difference between control and water stress was detectable 3–10 days after the beginning of stress depending on the genotype, while the effect of limited nitrogen supply only induced subtle phenotypic effects. Phenotypic traits showed different response dynamics as well as multiple and changing interaction patterns with stress progression. The estimated biovolume, leaf area index, and color ratios were found to be stress-responsive at different stages of drought stress progression and thereby represent valuable reference indicators in the selection of drought-adaptive genotypes. Furthermore, genotypes could be grouped according to two typical growth dynamic patterns in water stress treatments by c-means clustering analysis. Inbred lines with high drought adaptability across time and development were identified and could serve as a basis for designing novel genotypes with desired, stage-specific growth phenotypes under water stress through pyramiding. Drought recovery potential may play an equal role as drought tolerance in plant drought adaptation.

## Introduction

Crop research today is more important than ever as scientists confront global threats from climate changes and diseases which may affect food security and livelihoods of smallholder farmers around the world. Maize is one of the most important crops globally, and more than half of the increased food demand for cereal plants will come from maize (Yan et al., 2011). However, maize suffers from several production constraints of which limited water availability and nitrogen deficiency are the most restricting factors (Moser et al., 2006), frequently occurring together (Nyombayire et al., 2011). Average temperatures and water availability are predicted to become an increasing problem under future climate conditions (IPCC, 2014), which will also influence soil organic matter (Brevik, 2013). Thus, developing maize varieties with improved water and nutrient use efficiency is a primary breeding target (Fiorani and Schurr, 2013), for which efficient phenotyping approaches are necessary (Araus et al., 2012; Avramova et al., 2016; Zhang et al., 2017).

Automated phenotyping (a.k.a. phenomics) is widely regarded as a priority for future crop breeding research (Awada et al., 2018; Liang et al., 2018). Image-based phenotyping allows non-destructive analyses of dynamic processes in plant growth in response to diverse environmental conditions (Neilson et al., 2015; Ge et al., 2016; Muraya et al., 2017). Automated plant phenotyping contributes to the identification of a genetic variation to increase genetic gain within a breeding program (Araus et al., 2018). Previous studies showed that image-based traits are reliable estimators of manually measured traits (Humplík et al., 2015; Neilson et al., 2015) including high correlations between digital biovolume (biomass proxy) and measured yield (Chen et al., 2014; Honsdorf et al., 2014; Neumann et al., 2015; Ge et al., 2016; Muraya et al., 2017; Dodig et al., 2019). Although predicting field performances based on controlled environment experiments is still under question (Poorter et al., 2012; Araus and Cairns, 2014; Junker et al., 2015), there are several studies in the literature that report that glasshouse-grown potted plants could be extrapolated to field-grown plants (Kholová et al., 2012; Pardo et al., 2015; Peirone et al., 2018). In maize, it is shown that a considerably high percentage of the total variation in grain yield under drought conditions could be predicted by vegetative phenotypic data generated in water-limited controlled environments (Chapuis et al., 2012; Zhang et al., 2017).

The sensitivity of maize yield mainly varies over stressing periods in relation to growth stages, being the greatest at flowering in the case of drought (Vitale et al., 2009, 2011; Araus et al., 2012). Although the maize water requirement is highest in the reproductive stage, water shortage during the vegetative growth can also significantly reduce grain yield (Pandey et al., 2000; Çakir, 2004; Mi et al., 2018). Stress during the maize vegetative growth period leads to a significant reduction in plant height and leaf area (which determines radiation interception and biomass accumulation), which indirectly affect the growth rate at flowering and the seed number (Moser et al., 2006; Borras et al., 2009; Araus et al., 2012; Chapuis et al., 2012). Plant morphology is one of the most important types of phenotypic traits, feasible to provide access to every aspect of plant growth and stress response (Fahlgren et al., 2015; Wang et al., 2019). Plant growth was considered as a measure of stress-adaptive capacity (Dolferus, 2014), which integrated both stress tolerance and plant capability to resume growth after exposure to severe stress (Luo, 2010; Fang and Xiong, 2015). Research on early plant development can be used in plant breeding as an indirect measure of tolerance to abiotic stresses (Montes et al., 2011). Screening for drought tolerance of maize hybrids at the seedling stage under controlled conditions clearly demonstrates the potential to identify candidate drought-tolerant genotypes and reduce selection under field conditions (Avramova et al., 2016). Plants exposed to some kind of stress during the vegetative growth will often adapt their structure and physiology, which makes them more tolerant to the next limitation, at later stages of their life (Pieruschka and Schurr, 2019).

The life cycle of maize can be divided into vegetative (from emergence to tasseling) and reproductive (from silking to physiological maturity) phases (Ransom et al., 2014). Vegetative growth stages between emergence (VE) and tasseling (VT) are based usually upon the number of visible leaf collars (V stages). The period between V6 (six leaves with visible collars) and VT (tassel) is often referred to as the period of rapid growth. At VT, the plant has reached its full height and all leaves have emerged. While water stress prior to the V4 to V5 growth stages would probably cause little yield loss if plants survive, at V6, the development of tassels and ears starts, so water stress can cause more damage than at the seedling stage, with progressively more damage the closer the stress occurs to tasseling (Lee and Grove, 2012).

In the present study, we used a high-throughput platform to collect visible light images to identify genotypic differences and analyze and evaluate dynamic changes and developmental trends in 20 maize inbred lines (ILs) throughout the maize vegetative growth season. Data on biomass at different growth stages and from different genotypes can be used to form temporal profiles, which offer novel insights into the diversity of phenotype that could not be detected through traditional terminal measurements alone (Pugh et al., 2018; Han et al., 2019a). Detailed growth curves can provide new tools for plant breeders to assess overall plant growth and tolerance to various stresses throughout its development (Pauli et al., 2016). Furthermore, dynamic cluster analysis of time series data is of great significance since it could extract expression patterns and changing rules of traits in the time space dimension (Han et al., 2019b). Fuzzy c-means is one of the best-known clustering algorithms to organize the wide variety of datasets automatically and extract meaningful statistics. More specific objectives in this study were to identify (i) reliable and useful image-based traits for predicting biomass accumulation at different stages of stress progression, (ii) genotypic differences in dynamic changes and developmental trends of estimated biovolume (EBv) during different growth stages by time series clustering, and (iii) traits that contribute to drought adaptability (DAD) based on the relative growth of the studied ILs. In addition, the roles of drought tolerance (DTO) and drought recovery (DRC) in DAD of maize were comparatively analyzed.

## Materials and Methods

### Plant Material

Twenty temperate maize (*Zea mays* L.) ILs representing a set of public and commercial inbreds with different tolerance to abiotic (mainly drought) stresses were selected for the experiment (Dodig et al., 2019). The developmental or collection origin of the 20 ILs used in this study and further information such as the maturity group and the germplasm pool they belong to are given in Supplementary Table 1. All seeds used in this study were obtained from the Maize Research Institute gene bank, Zemun Polje, Serbia, and multiplied in a single year under non-stress conditions.

### Growth Conditions

A pilot study was performed in greenhouse conditions to ensure phenological synchronization across 20 ILs at the targeted stage when nitrogen and water stress were to be imposed. Information on the number of days to the six-leaf stage was used as covariate adjustment to group genotypes into subsets of similar phenology (early, intermediate, and late) for sowing at different times (Dodig et al., 2019). Plants were transplanted and entered the IPK automated platform in 5.5 L pots filled with the IPK soil mixture (Junker et al., 2015). The temperature regime during the experiment was set to mimic the Zemun Polje vegetative temperature, which raised stepwise sequentially during the growth period starting with 20/15°C day/night during germination and the preculture period, then 22/17°C day/night for 10 days, and finally to 25/20°C day/night temperature for further 25 days. During the entire cultivation period, relative air humidity was set to a minimum of 65% and the light period was set to 16 h (06:00–22:00 h) with an average total illumination of approx. 350 mmol m^–2^ s^–1^ PAR. For more details on growth conditions during germination, preculture, and culture period, see Dodig et al. (2019).

### Experimental Design and Phenotyping

The lines were phenotyped using the automated non-invasive plant phenotyping system for large plants (Junker et al., 2015) at the Leibniz Institute of Plant Genetics and Crop Plant Research (IPK), Gatersleben, Germany. Plants were grown in a greenhouse using a setup that aimed at controlling the amount of both nutrient and water while trying to best mimic natural conditions. In brief, after the precultivation period, plants were transplanted in larger pots [at the stage of four to five leaf count (LCn) across ILs] and transferred into the IPK automated plant phenotyping platform, where ILs were grown for further 35 days. Plants were exposed to control (C) and limiting nitrogen (N) and water conditions (W), as well as to the combination of NW. Optimal and limiting water conditions were defined as 75 and 30% of soil field capacity (SFC), respectively, while optimal and limiting nitrogen conditions were defined as 35 and 15 mg of nitrogen per pot, respectively. The control treatment involved optimal water and nitrogen supplies. In W and NW treatments, water stress was initiated at 9 days after transplanting (DAT) by cumulative soil drying to 20% SFC (DAT 22) and then raised to 30% SFC and kept at this level until the end of the experiment (Supplementary Figure 1). In the C and N treatments, pots were watered daily to a target weight corresponding to 75% SFC from transplanting (DAT 0) to DAT 35. SFC in all pots and each treatment was adjusted to the same level based on daily measurements of pot weight for all ILs (Junker et al., 2015). In N and NW treatments, nitrogen stress was initiated at DAT 8 by applying reduced nitrogen level compared with control and repeated in three more occasions till the end of the experiment (Supplementary Figure 1). In total, 140 mg of nitrogen per pot (equivalent to 25.5 mg L^–1^ substrate) was applied in optimal nitrogen conditions and 60 mg of nitrogen per pot (equivalent to 10.9 mg L^–1^ substrate) in reduced nitrogen conditions during 4 weeks. More details on fertilizer type used are given in Dodig et al. (2019).

In each treatment, eight plants per IL were tested, which resulted in a total of 640 plants. Plants were phenotyped from DAT 2 (five LCn across ILs) to DAT 35 (12 LCn across ILs) at 33 time points. Technical issues resulted in a loss of images for DAT 30, although watering continued. At each time point, four side-view images and one top-view image of each plant were taken at imaging boxes for acquiring visible light (RGB), static fluorescence (FLUO), and near-infrared image data. The IPK Integrated Analysis Platform (IAP version 0.9; Klukas et al., 2014) was used for the image (pre-) processing and automated feature extraction. The majority of the used traits in this study were derived from RGB images, except one FLUO-based trait (Supplementary Table 2). The experimental design and phenotyping are described in more detail in Dodig et al. (2019).

### Image-Derived Plant Traits and Indices

To assess maize growth over time, the following 12 different image-derived plant traits were chosen to be analyzed in this study: EBv, side compactness (SCom), solidity (Sol), surface coverage (SCov), caliper length (CLe), plent height (PHg), LCn, yellow to green (Y2G) ratio, blue to green (B2G) ratio, lab color a (Lab_a), lab color b (Lab_b), and static fluorescence intensity (FI). Selected image-derived traits could be broadly classified into biomass-related (EBv), architectural (SCom, Sol, SCov, CLe, PHg, and Ln), and physiological (Y2G, B2G, Lab_a, Lab_b, and FI) traits. The detailed information for image-based trait definitions and details of the trait extraction are shown in Supplementary Table 2. EBv showed to be a very good proxy of biomass in our experiment (Dodig et al., 2019). The majority of the selected traits were also substantially important for vegetative fresh and/or dry biomass weight in one or all treatments based on LASSO and Ridge regression models (Dodig et al., 2019). FI showed not to be indicative of the vegetative plant performance at a single time point (harvest) in the previous study by the same authors. However, it is included in this study to be checked at multiple time points, since it is widely used in plant physiology research. PHg is added to the list as this trait is regarded to be a key contributor to maize biomass yield. In addition, LCn is included into the list as a key trait to describe plant phenology. Based on the EBv, a relative growth rate was calculated for each treatment. The relative growth rate (voxel day^–1^) was calculated as RGR = (Ln W2 − Ln W1)/(T2 − T1), where W1 and W2 are plant EBvs at times T1 and T2, while Ln is a natural logarithm. To estimate genetic variability and roles of DTO and DRC in drought adaptation, for each IL, DTO, DRC, and DAD were estimated based on the relative growth of EBv during drought stress (DTO = DAT 22_treatment_ − DAT 9_treatment_/DAT 22_control_ − DAT 9_control_), rewatering (DRC = DAT 35_treatment_ − DAT 23_treatment_/DAT 35_control_ − DAT 23_control_) and the entire water stress cycle (DAD = DAT 35_treatment_ − DAT 9_treatment_/DAT 35_control_ − DAT 9_control_), respectively. DAT 9, DAT 22, DAT 23, and DAT 35 refer to day of stress initiation, day of maximum stress, start of rewatering, and day of harvest (end of the experiment), respectively. Water use efficiency based on EBv and water applied (voxel g^–1^ water) was calculated for each IL during drought stress (EBv_DAT 22_ − EBv_DAT 2_/water applied) and recovery (EBv_DAT 35_ − EBv_DAT 22_/water applied) separately.

### Statistical Analysis

To analyze the degree of variation during the different time points, EBv from data collected on 20 ILs was plotted against the coefficient of variation (CV, %). CV was calculated with mean and standard deviation. Restricted maximum likelihood (REML) was conducted to determine the overall importance of the factors like genotype, treatment, and their interaction for EBv over time. The analysis follows a Gaussian linear mixed model which was formulated for each day separately. The effect of replication in the model was treated as fixed, while the effects of genotype, treatment, and the interaction were treated as random effects. Diagrams with error bars were used to present the development of image-derived traits and relative growth rate in each treatment over time. The diagram *y*-axis was generated by the means of all ILs, while the *x*-axis represents DAT. At each DAT, data were analyzed by analyses of variance (ANOVA linear mixed model) to test the effect of treatment using Tukey’s *post hoc* test, and a *P* < 0.05 was considered significant. The analysis of covariance structure was done using the linear mixed model (Gilsum et al., 2009). Five different covariance structures of the observations within the same image-based trait in each treatment were compared: uniform (UN), power (POWER), heterogeneous power (HETPOWER), antedependence (ANTE), and unstructured (US). The Bayesian information criterion (BIC) value was used to find the best matrix among each model considered, so that the lower its value, the better the adjustment of the model in question.

To identify the trait mean dynamic change pattern, cluster analysis was applied on a time series dataset for EBv using the fuzzy c-means clustering algorithm (Han et al., 2019b). The best number of clusters is determined by using the majority rule (Charrad et al., 2014). −Log10 (*P*-value) was used for the differential expressions between obtained cluster groups. After clustering, a typical curve is generated by connecting the mean of EBv data during the period between the maximum growth before maximum water stress and the day following maximum water stress (from DAT 19 to DAT 23) for each cluster. A typical curve of EBv represents a group of genotypes that had similar dynamic change.

Pearson linear correlations were calculated to analyze the dynamic changes of the relationship between EBv and other studied traits over time. Simple linear correlations were also used to determine the relationship among drought-adaptive capabilities of the various genotypes and the relationship between their drought-adaptive capabilities and their image-derived traits and relative growth rate at two time points. Statistical analysis and data visualization were completed using the R software (R Core Team, 2016) and Microsoft Excel (Office 2010). The mixed model analyses were conducted with the ASReml software (Gilmour et al., 2009). The analysis of covariance structure was conducted using the mixed model procedures of SAS 9.3 version (SAS Institute, 2011).

## Results

### Phenology

Image-derived LCn, as a proxy of leaf developmental stage (V), was used to compare the phenology of ILs and treatments during the imaging period (Supplementary Tables 3–6). At the time of the beginning of imaging (DAT 2), plants had approximately five to six leaves in all treatments. At the time of nitrogen and water stress imposed (DAT 8 and DAT 9, respectively), plants had approximately seven to eight leaves in all treatments. At the time of maximum water stress (DAT 22), plants had from 7–11 leaves in NW, over 8–11 leaves in W, 9–11 leaves in N, to 9–12 leaves in C. At the end of the experiment (DAT 35), plants had approximately 11–14 leaves (in C and N) and 8–13 leaves (in N and NW) at the developmental stage. Two ILs reached the VT developmental stage (visually assessed) in C and N (ILs 13 and 19), while two ILs reached the VT developmental stage in all treatments (ILs 8 and 11). On average, the N treatment did not slow down the leaf development rate compared with C as in both treatments the average LCn was 13 at DAT 35. However, in W and NW treatments, the mean leaf developmental stage was by two and three leaves less compared with C and N, respectively.

### Dynamic Treatment Effect on Image-Derived Traits and the Relative Growth Rate

**FIGURE 1:**
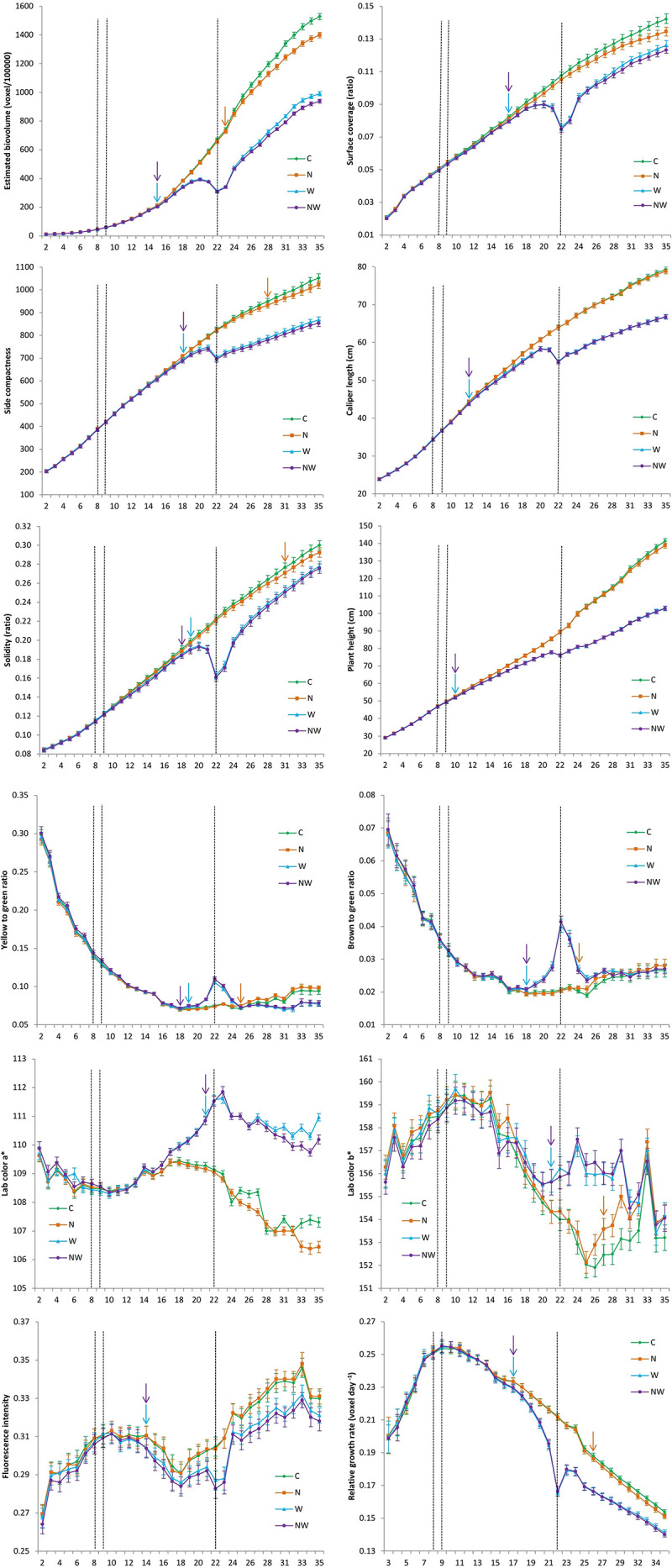
Development of image-derived morphological and colour traits as well as relative growth rate in control (C), nitrogen stress (N), water stress (W) and combined nitrogen and water stress (NW) conditions across inbred lines over time. Data points were missing on DAT 30. The *x*-axis represents days after transplanting. Vertical bars represent standard error of the mean. Vertical dashed lines represent the beginning of both nitrogen stress (DAT 8) and water stress (DAT 9), and maximum stress recorded when soil water capacity (SFC) was at the minimum of 20% (DAT 22). Vertical arrows represent time points at which difference between treatment and control become significant (*P* ≤ 0.05). SFC gradually declined in the period from DAT 2 (75%) to DAT 22 (20%), and then was slightly increasing to 30% until the end of the experiment (DAT 35).

The temporal dynamics of the influence of treatment on the overall image-derived trait and RGR development based on daily non-invasive imaging across ILs are shown in Figure 1, as well as in Supplementary Table 7. Lower water availability in W and NW compared with C had a significant negative effect on PHg (starting from DAT 12), EBv (DAT 15), SCom (DAT 18), SCov (DAT 19), CLe (DAT 19), and Sol (DAT 20) development, and all traits continually significantly reduced until the end of the experiment. Due to less severe nitrogen stress than water stress, its effect on morphological image-derived trait development was significant in comparison with C later in the course of the trial: EBv (starting from DAT 23), SCom (from DAT 28), and Sol (from DAT 31). The low N effect on SCov, CLe, and PHg was not reached by the end of the experiment. In NW, all morphological image-derived traits (except CLe) were significantly reduced compared with water stress only (W) from DAT 22 (EBv), DAT 26 (SCom), DAT 28 (PHg), and DAT 34 (Sol and SCov) to the end of the experiment. The most sensitive image-derived morphological trait to water and combined water and nitrogen stress was EBv with the maximum reduction of 53 and 54% at DAT 22 in W and NW compared with C, respectively (Supplementary Table 7). EBv was also the most reduced trait in N of 8% at DAT 35 compared with C, followed by SCov (5%). Cle was the most tolerant trait with maximum reduction of 14% (DAT 22) in both W and NW.

Among color-related traits, B2G and Y2G were the most sensitive to water stress. A significant increase in B2G and Y2G ratios in W and NW compared with C was observed 10 and 11 days after stress was applied, and was significant until DAT 28 and DAT 24, respectively (Supplementary Table 7). However, at the end of the experiment, Y2G was significantly higher in C compared with W and NW, probably due to a phenomenon called physiological leaf spots (Neumann et al., 2015). Regarding N treatment, a significant increase in B2G and Y2G ratios compared with C was observed at DAT 24 and DAT 25, respectively, and was significant until the end of the experiment. Other two color traits, Lab_a and Lab_b, showed lower variability and less sensitivity to the applied stresses than color ratios. A significant increase in both Lab_a and Lab_b was observed only in water stress treatments at DAT 21 and remained significant until DAT 31 and DAT 35, respectively. A significant difference of FI in water stress treatments compared with control started at 14 DAT and continued until the end of the experiment; in N, there was no significant difference with C at any time point. RGR was significantly lower in water stress treatments and N compared with C from DAT 17 to DAT 35 and from DAT 26 to DAT 35, respectively.

### Phenotypic and Genotypic Biovolume Variation Over Time

**FIGURE 2:**
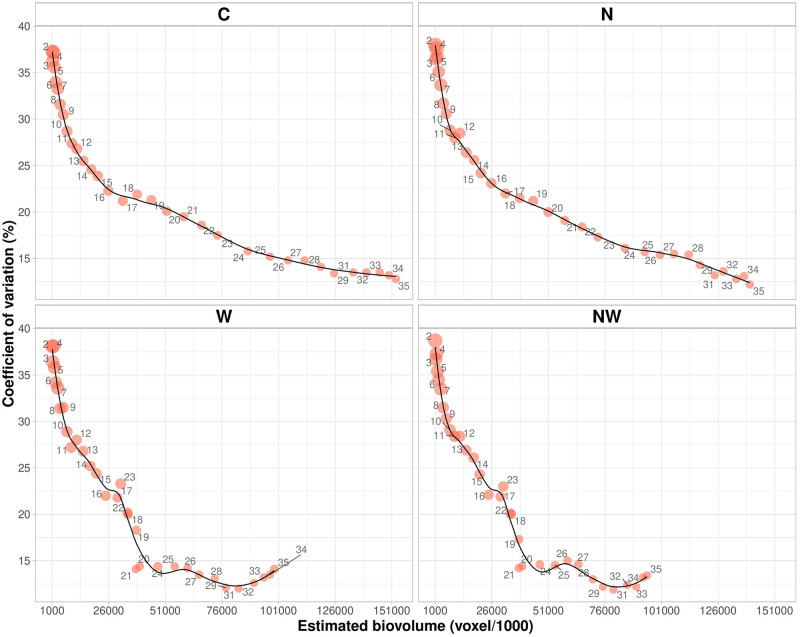
Estimated biovolume plotted against the coefficient of variation over time (DAT 2–DAT 35) from data collected on 20 inbred lines in control (C), nitrogen stress (N), water stress (W), and combined nitrogen and water stress (NW) conditions. A circle represents a ratio of the minimum to the maximum. Data points were missing on DAT 30.

The phenotypic variation of EBv with plant growth and the distribution of the respective CV (%) in different treatments over time are given in Figure 2. In C and N treatments, the degree of variation of EBv at earlier growth stages (from DAT 2 to DAT 13–14) was very high (more than 35%) with continuous decrease in the rest of the period. At the end of the experiment, variation of EBv in C and N was moderate and the lowest for the entire period (12.8 and 9.9%, respectively). In W and NW, the degree of variation of EBv was also very high of over 35% at earlier growth stages. After the period of EBv decreasing from DAT 14 (24–25%) to DAT 21 (13–14%), it shortly increased (20–23%) in the period of maximum stress (DAT 22–23) and then again dropped to the moderate level (12–13%) until the end of the experiment.

**FIGURE 3:**
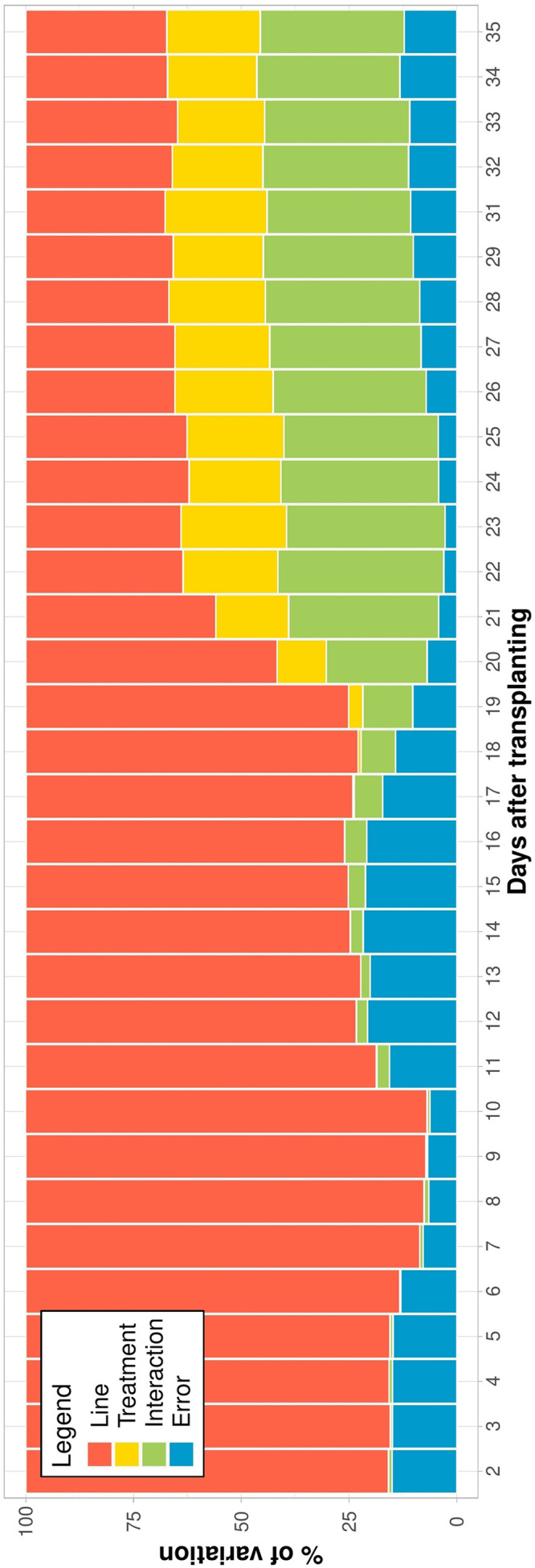
The percentage of estimated biovolume variation explained by components of variance for each day of imaging (shown as the number of days after transplanting—DAT) across inbred lines and treatments. Data points were missing on DAT 30.

To capture genotypic differences among ILs over time, the relative contribution of the variance components to the phenotypic variance of EBv was determined. Within each treatment, the variation of EBv explained by the genotype was consistently high (over 80% in N and over 90% in C, W, and NW) throughout the growing experimental period (Supplementary Figure 2). However, the analysis across treatments showed that as stress got higher the genetic variation got lower and the interaction term became more prominent (Figure 3). In the early stage of the experiment, until shortly after the initiation of stresses (DAT 10), the variation explained by the genotype was high (over 80%). From then, the proportion of phenotypic variance of EBv attributable to genetic variance was gradually decreasing, and the genotype × treatment interaction was steadily increasing until maximum stress (being 37 and 39% at DAT 22, respectively). From DAT 22 to the end of the experiment, the variation explained by the genotype and the interaction was constant and similar of about 32–38%, followed by the treatment effect (20–25%) and the residual variation (2–14%).

### Covariance Structure of Image-Based Traits Over Time

Analysis of the structure of covariance was conducted to explain the response of variability and correlations between the repeated measurements. Five covariance structures of the observations within the same image-based trait in each treatment were analyzed. The BIC value was used to find the best covariance structure, so that the lower its value, the better the adjustment of the model in question (presented in Supplementary Table 8). The most appropriate covariance structure for EBv, SCov, and Sol was HEATPOW. For other morphological image-derived traits SCom, CLe, and PHg, the POWER structure was found to be the best in most treatments. ANTE gave the best fit to describe covariance structure of color-related traits and FI over time.

### Change of Phenotypic Correlations Between Digital Biomass and Other Measured Traits

**FIGURE 4:**
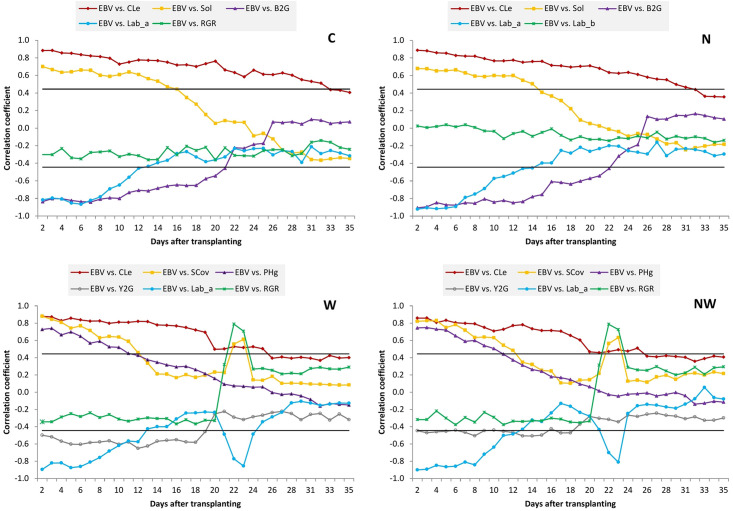
Pearson’s correlation coefficients between estimated biovolume and other image-derived traits over time. Only examples of the typical point and figure trend line for each treatment are presented. C, control treatment; N, nitrogen stress treatment; W, water stress treatment; NW, combined nitrogen and water stress treatment. Horizontal lines represent the critical value of the correlation coefficient at *P* ≤ 0.05.

The patterns of trait correlations over time from DAT 2 to DAT 35 in each treatment are presented in Supplementary Tables 9–12. Nearly all associations had a similar pattern over time in different treatments. Nevertheless, there were several associations that showed different patterns between non-stress and stressed treatments. Figure 4 shows a sample of typical correlation patterns for each treatment. In general, phenotypic correlations between EBv and other image-derived traits at early stages are much higher than those at later stages. All morphological traits at early stages had a strong positive correlation with EBv in all treatments, but the correlation coefficient decreased until the end of the experiment, remaining positive or eventually getting slightly negative. The longest duration of significant correlation was recorded for the EBv–CLe association (from DAT 2 to DAT 25 in N and NW, i.e., to DAT 31–33 in N and C, respectively). In water stress treatments (N and NW) after DAT 22 (maximum water stress), only the EBv–CLe association was significant. In some cases (e.g., EBv–SCov and EBv–Sol), associations re-established the significance around maximum stress (DAT 22–23) and then again became non-significant until the end of the experiment.

Color ratios Y2G and B2G had a similar pattern of correlations over time with EBv. They had strong negative correlations with EBv at early stages (up to DAT 18–23 across treatments), and then the correlation coefficient decreased until the end of experiment, remaining negative (N and NW) or even got slightly positive (C and N). However, while the EBv–Y2G association was not significant in the period shortly before and after maximum stress at DAT 22, the EBv–B2G association remains significant and moderately negative (∼0.45–0.50). Lab_a had a similar correlation pattern with EBv as two color ratios in C and N, while Lab_b in these treatments had a low and non-significant correlation with EBv during the whole imaging period. In water stress treatments, the EBv–Lab_a association was significant only at the beginning, but it re-established its significance around maximum stress (DAT 21–23). A significant (moderate) correlation between EBv and Lab_b in W and NW treatments was established only during and shortly after maximum stress (DAT 22–24).

Fluorescence intensity had no significant correlation with EBv at any day in the control treatment. In N, although the EBv–FI association was increasing until the end of the experiment to be moderately positive, it remained non-significant. It is possible that in case of continuing N stress, it will become significant. However, in W and NW, the EBv–FI association was moderately positive and significant around maximum stress and shortly after that (DAT 22–29). Finally, RGR had no effect on EBv over time in the C and N treatments. However, in a very short period around maximum stress (DAT 22–23) in W and NW treatments, RGR established a very strong (0.70–0.80) and positive correlation with EBv.

### Clustering of Temporal Biomass Accumulation in ILs Under Different Stress Regimes

**FIGURE 5:**
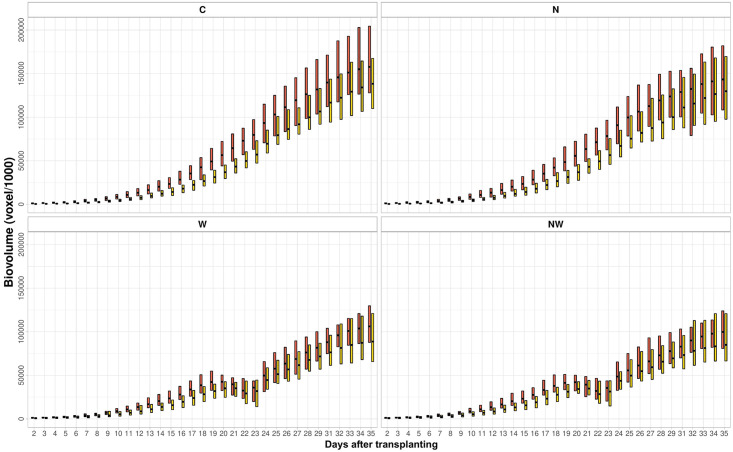
Clustering of temporal profiles of estimated biovolume. Temporal profile of each inbred line is assigned to group A or B by fuzzy c-means clustering. The box plot represents the full range of variation (from minimum to maximum) and mean value. C, control; N, nitrogen stress; W, water stress; NW, combined nitrogen and water stress. Data points were missing on DAT 30.

To investigate whether ILs can express EBv in similar patterns, temporal profiles were clustered by using the fuzzy c-means clustering algorithm. For clustering temporal profiles of EBv, we obtained an optimal number of two (hereafter named as groups A and B) typical dynamic patterns for each treatment, but of a different size (Supplementary Figure 3). Temporal patterns of EBv in 20 maize ILs in groups A and B within treatments are presented in Figure 5. The upward trends of EBv profiles observed in groups A (15 temporal profiles) and B (five temporal profiles) in both C and N were similar. However, cluster A consistently showed a significantly higher EBv than cluster B in both treatments (Supplementary Tables 13, 14). The difference in EBv between group A and group B was more expressed in the earlier stages of growth than in the later stages, being more than double reduced from DAT 2 to DAT 35. The difference between A and B in EBv was constantly significant, but the level of significance was decreasing toward the end of the experiment, particularly in N. The average values of morphological image-derived traits were lower in group B than in group A through the entire period, except for Sol and SCov, which were higher in group B than in group A after maximum stress had been reached (Supplementary Tables 17, 18). RGR was constantly higher in group B than in group A during the whole imaging period in both C and N. The difference in Y2G and B2G between the two groups was higher at earlier stages of growth (in favor of group B) than toward the end of the experiment. Values of other color-related traits (Lab_a and Lab_b) as well as FI were similar for both groups across all imaging periods in C and N.

**FIGURE 6:**
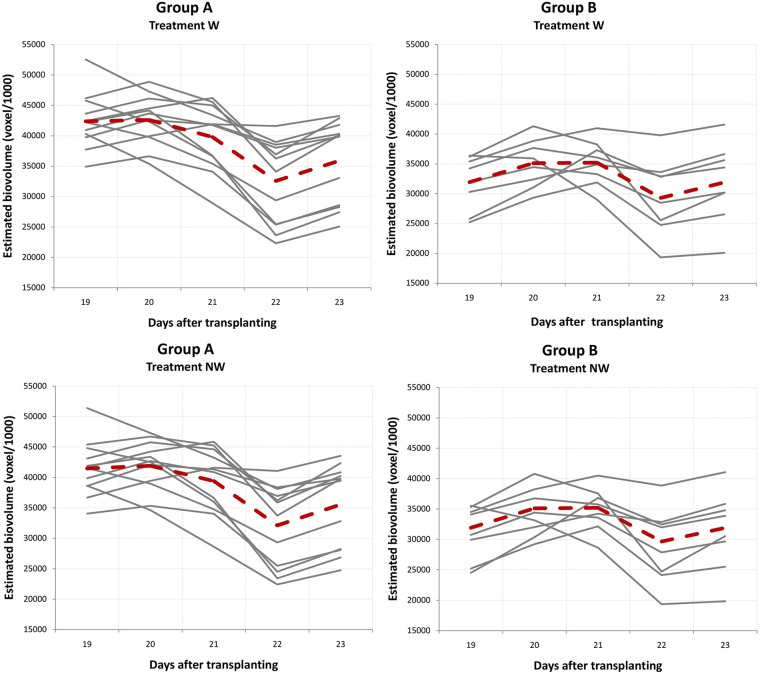
Generated typical curve for the period between the maximum growth before maximum stress and the day following maximum stress for groups A and B obtained by fuzzy c-means clustering. Inbred lines reached their maximum growth between DAT 19 and DAT 21, with the bottom level recorded for all inbred lines at DAT 22 (maximum stress). Dashed lines show a polyline formed joining the clustering means at five time points. W, water stress; NW, combined nitrogen and water stress.

Due to water stress, the temporal patterns of EBv accumulation were not linear with time and demonstrated the upward and the downward variation in W and NW (Supplementary Tables 15, 16). Group A (12 temporal profiles) consistently showed a significantly higher EBv than group B (eight temporal profiles) in both water stress treatments. The differences in EBv between groups A and B were more expressed in the earlier stages of growth than in the later stages of growth. All ILs reached their minimum EBv at maximum stress (DAT 22) and then continued to gradually increase after rewatering (from SFC 20% to SFC 30%) until the end of the experiment (DAT 35). However, several ILs showed at an earlier time and a more rapid decline immediately following maximum growth before DAT 22. Namely, the EBv decline across ILs took from 1 to 3 days following maximum growth prior to maximum stress at DAT 22 (Supplementary Tables 15, 16). The observed decline ranged, more or less, from 1% (IL 16) to 45–46% (ILs 4, 11, and 14) in both W and NW. The typical temporal profile of groups A and B for the period between DAT 19 and DAT 23 (period between maximum growth before maximum stress and the day following maximum stress) is presented in Figure 6. Both groups A and B had an increase in EBv from DAT 19 to DAT 20 by about 1 and 10% in both N and NW, respectively. While the EBv of group A started to decrease at DAT 20, the EBv of group B continued to increase up to DAT 21. Finally, both groups reached the bottom level at DAT 22 and then started to grow up again as SFC increased from 20 to 30% by watering done after imaging at DAT 22. On average, the EBv decline immediately following the maximum growth before DAT 22 in water stress treatments was higher in group A (23%) than in group B (16–17%). RGR had a similar decline in groups A and B in W and NW during the mentioned period (26 vs. 18–20%, respectively). The patterns of difference between group A and group B for RGR and other image-derived traits in W and NW were similar to those in C and N (Supplementary Tables 19, 20).

### Drought Tolerance, Recovery, and Adaptability of ILs

**FIGURE 7:**
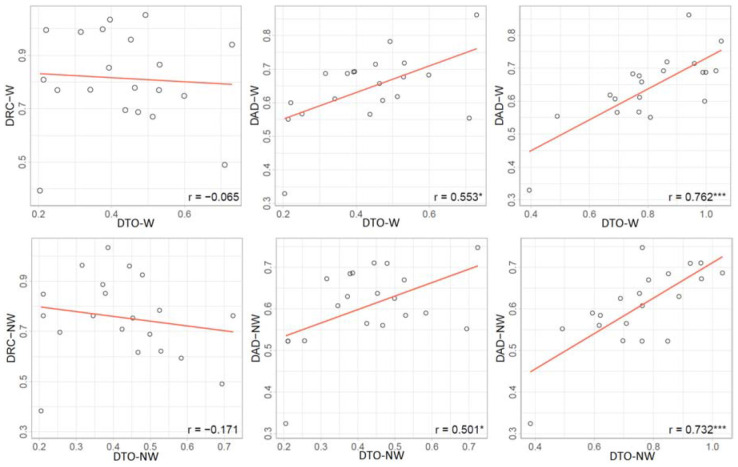
Correlations between drought-adaptive capabilities in water stress (W) and combined nitrogen and water stress (NW). DTO, drought tolerance; DRC, drought recovery; DAD, drought adaptability. Asterisks indicate significant differences between drought-adaptive capabilities (*P ≤ 0.05; ***P ≤ 0.001).

Drought tolerance, DRC, and DAD were estimated in water stress treatments based on the relative growth of EBv during drought stress (DAT 9–22), rewatering (DAT 23–35), and the entire cycle (DAT 9–35), respectively. DTO, DRC, and DAD (hereinafter referred to as drought-adaptive capabilities) showed a substantial variation among the ILs (Supplementary Table 21). IL 8 showed the strongest DTO at up to 0.730 (W) and 0.724 (NW), while IL 4 showed the weakest at 0.204 (W) and 0.205 (NW). IL 19 had the strongest DRC over all water stress treatments (ranked 2 and 1 in W and NW, respectively), while IL 4 showed the weakest DRC in water stress treatments. Plant DAD was the strongest in ILs 8 and 6, while it was the weakest in ILs 4 and 14. On average, group A had a statistically weaker DTO but stronger DRC than group B (Supplementary Table 21). Furthermore, group A showed stronger, although not significant, DAD compared with group B in both water stress treatments. DAD can result from either strong tolerance or strong recovery. DAD had a higher correlation with DRC in W (*r* = 0.762^∗∗∗^) and NW (*r* = 0.733^∗∗∗^) than with DTO (*r* = 0.552^∗^ and 0.499^∗^ in W and NW, respectively) (Figure 7). There was little correlation (*r* = −0.065 and −0.171 in W and NW, respectively) between DTO and DRC. DTO was in a significant and positive relationship with WUE and RGR, but not with plant size in both water-limiting treatments. On the other side, DRC was in a significant and positive relationship with WUE and plant size (except plant size in NW), but not with RGR (Supplementary Figure 4).

### Relationship Between Image-Derived Traits and Drought-Adaptive Capabilities

To define which image-derived traits may contribute to plant drought adaptation, correlation analysis between drought-adaptive capabilities and image-derived traits was conducted at two time points in W and NW (Table 1). At the time of maximum water stress (DAT 22), SCov, Sol, EBv, and RGR had a significant positive correlation with DTO and DAD in both treatments, while Y2G, B2G, and Lab_a (only in NW) had a significant negative correlation with DTO. There was no significant correlation between DRC and image-derived traits in any of the treatments at DAT 22. At the end of the experiment (after recovery, DAT 35), the only significant correlations in W and NW found were positive correlations between RGR and DTO, RGR and DAD, EBv and DRC, EBv and DAD, and SCom and DRC (only in NW).

**TABLE 1:** Correlations (*r*) between drought-adaptive capabilities and image-based traits at the maximum stress (DAT 22) and after recovery (DAT 35) under water stress and combined nitrogen and water stress.

**Trait**	**DAT 22**			**DAT 35**		
	**DTO**	**DRC**	**DAD**	**DTO**	**DRC**	**DAD**
**Water stress**						
SCom	0.28	–0.27	0.02	0.36	–0.07	0.25
Sol	0.66**	0.40	0.70***	0.01	0.38	0.34
SCov	0.71***	0.31	0.66**	–0.18	0.32	0.15
CLe	–0.10	0.33	0.13	0.06	0.18	0.16
PHg	–0.17	–0.24	–0.38	–0.14	–0.30	–0.34
LCn	0.01	0.12	0.12	0.10	0.04	0.20
EBv	0.65**	0.40	0.65**	0.03	0.74***	0.66**
FI	0.16	0.16	0.16	0.09	0.26	0.21
Y2G	−0.50*	0.03	–0.24	0.10	–0.21	–0.11
B2G	−0.60**	0.03	–0.28	–0.19	0.17	0.07
Lab_a	0.07	0.04	0.10	0.10	0.07	0.14
Lab_b	0.13	0.23	0.20	0.06	0.27	0.21
RGR	0.83***	0.19	0.70***	0.60**	0.20	0.62**
**Combined nitrogen and water stress**						
SCom	0.35	–0.20	0.13	0.34	–0.08	0.23
Sol	0.66**	0.31	0.65**	0.00	0.31	0.65**
SCov	0.71***	0.14	0.54*	–0.12	0.47*	0.35
CLe	–0.11	0.37	0.17	0.06	0.19	0.18
PHg	–0.18	–0.11	–0.29	–0.12	–0.23	–0.29
LCn	0.04	0.25	0.37	0.13	0.04	0.24
EBv	0.64**	0.28	0.58**	–0.05	0.73***	0.63**
FI	0.21	0.11	0.17	–0.06	0.05	–0.04
Y2G	−0.59**	0.02	–0.36	–0.11	–0.13	–0.26
B2G	−0.65**	0.06	–0.35	–0.36	0.01	–0.25
Lab_a	0.67**	–0.10	–0.44	–0.37	0.15	0.13
Lab_b	0.22	0.24	0.30	0.06	0.23	0.20
RGR	0.48*	0.19	0.68***	0.57**	0.29	0.70***

DAT, days after transplanting; DTO, drought tolerance; DRC, drought recovery; DAD, drought adaptability. *P ≤ 0.05, **P ≤ 0.01, ***P ≤ 0.001.

## Discussion

To keep up with the increasing food demand, breeders aim to measure a large number of plants in various stress conditions, like drought or low fertility soils, to identify traits that will make the plants more tolerant to a changing climate and to identify the best progeny. High-throughput phenotyping techniques based on sensor technology are considered as important tools to better understand the interaction of a genotype with the environment for rapid advancement of genetic gain in breeding programs (Zhao et al., 2019). The aim of this study was to assess the genetic variation of growth dynamics in 20 maize ILs through automated non-invasive phenotyping based on visible light (RGB) imaging in different stress conditions. This allowed us to assess the stress response dynamics across young developmental stages and go in line with the report about existing reliable genetic factors to the early- and the mid-season growth in maize that cannot be captured by conventional measurements at the end of growth (Muraya et al., 2017; Pugh et al., 2018). Research on early plant development is a common practice in plants demonstrating the potential to identify stress-tolerant genotypes and reduce selection under field conditions (Montes et al., 2011; Avramova et al., 2016).

Genotypic differences were identified for 13 biomass-related morphological and physiological image-derived traits. The dynamic growth phenotype was measured from LCn of 5–6 (all treatments) to 8–13 (N and NW) and 11–14 (C and N) across 20 ILs with 33 time points each. The difference in the development stages among ILs was continuously increasing during the experiment in each treatment, being more prominent in water stress treatments. However, at the time of NW initiation (DAT 8 and DAT 9, respectively), the difference between the earliest and latest IL was still reasonably low of only one leaf difference. Additionally, the majority of the ILs (18 out of 20 and 17 out of 20 in W and NW, respectively) were within only one leaf difference (8–9) at the maximum water stress (DAT 22) that we can consider phenologies to be similar to reduce likelihood of artifacts.

The EBv showed a good correlation with the manually measured fresh weight and dry weight (Dodig et al., 2019) and, thus, can be used to represent the biomass. Maize plants in C and N treatments showed a continuous increase in the EBv and other image-derived morphological traits through the period of the imaging. Maize growth is reported to be logarithmic up to or slightly past flowering (Pugh et al., 2018). Although our intention was to obtain moderately reduced nitrogen growth conditions, the applied nitrogen stress appeared to be in a mild and more chronic fashion. This resulted in a less observable phenotypic effect in the N treatment compared with W and NW treatments. For some traits such as SCov, CLe, and PHg, a significant N effect was not reached by the end of the experiment. On the other hand, the applied water stress level built up rapidly and was quite severe, particularly when SFC was between 30 and 20%. All genotypes after the first increase (from DAT 2 to DAT 18) showed a decrease in the EBv (from DAT 19 to DAT 22), yet the time and quantity of the adverse effects of water stress on the growth varied among the inbreds (this will be discussed later). All ILs showed wilting symptoms from DAT 19 to DAT 22, and consequently, plant size (height and width) and leaf area were decreasing. Under water deficit conditions, plants exhibited several important physiological responses, including decreased cell turgor (Chaves et al., 2009) and leaf rolling (Sudhakar et al., 2016). Along with size, a lower turgor pressure results in a “wilted” cell or plant structure (i.e., leaf and stalk). The change in the leaf shape or form (leaf rolling) of plants experiencing water deficit provides a less exposed surface area to reduce transpiration. Both decreased cell turgor and leaf rolling led to the decreasing number of plant pixels on RGB images at the time of maximum stress. At the same time, physiological traits, such as color ratios of Y2G and brown to green, which may represent stress or wilting symptoms (Neumann et al., 2015), were at the maximum at DAT 22. Especially color traits were identified as early response traits which represent signs of wilting before the permanent wilting point is reached. At the permanent wilting point (commonly estimated for agricultural crops at −1.5 kPa soil matric potential), plants fail to recover their turgor upon rewetting (Tolk, 2003). In our case, maize plants experienced temporary wilting symptoms as they all recovered after increasing SFC from 20 to 30%.

The coefficient of variation was used to analyze the degree of phenotypic EBv variation over time. The change in the coefficient of variation may reflect the variation in the traits with crop growth (Han et al., 2018). In C and N treatments, heterogeneity of the EBv among ILs gradually decreased, which may suggest that the function of the EBv for plant growth is better during early growth stages than later ones. This is in agreement with Han et al. (2018) who analyzed the phenotypic variation of PHg and canopy cover during four critical growth stages of maize in non-stress field conditions. However, during severe (maximum) stress in W and NW, ILs became more different, which is expected as studied ILs are mixture of resistant and susceptible ones. Increased genetic variability in drought conditions is commonly observed, e.g., in maize (Campos et al., 2004). Observed genotypic variation of the EBv was consistently high throughout the growing period in each treatment. However, variation of the EBv explained by the genotype across treatments showed a decreasing genetic variation as stress get higher and the interaction term became equal to the genetic term. Our results suggests that the variation in the EBv during the plant growth is largely governed by genetic and interaction factors, rather than the treatment factor. Significant genotype × treatment effects indicate that genetically determined differences in the stress response were high enough to be detected by the EBv trait. Crop external phenotypes, such as morphology or biomass, results from internal phenotypes (structural traits from the cellular to the plant level and physiological traits), which are also influenced by the genotype × environment interaction (Zhao et al., 2019).

Generally, correlations between the EBv and image-derived morphological and color-related traits were much better at earlier than later growth stages. The phenomenon that correlations between various maize plant growth parameters became weaker at later growth stages was also reported by other authors in field (Freeman et al., 2007) and controlled conditions (Ge et al., 2016; Muraya et al., 2017). This can be attributed to the large difference in the plant architecture of maize genotypes as plants grow larger (Ge et al., 2016). In addition, the correct determination of some image-derived traits at later developmental stages can be biased by overlapping leaves or by reduced accuracy of measurements on material that are too tall (Golzarian et al., 2011; Pereyra-Irujo et al., 2012; Humplík et al., 2015; Ge et al., 2016; Pugh et al., 2018). Related to these, covariance structures that best explain the response of variability and correlations between the repeated measurements showed that correlations either decay (EBv and morphological traits) or change (FI and color-related traits) over days.

In the control condition, CLe and PHg (i.e., plant size) were two of the traits that had the longest duration of a significant correlation with the EBv. Both traits may be regarded as important traits in maize because they are highly heritable (Peiffer et al., 2014; Dodig et al., 2019) and easy to measure. It was reported that PHg correlated highly with biomass or grain yield in maize (Salas Fernandez et al., 2009; Yin et al., 2011; Barrero Farfan et al., 2013; Han et al., 2019b). Caliper length was a significant contributor to manually measured fresh and dry weights in maize grown under non-stress conditions (Dodig et al., 2019). A longer caliper was consistent with a larger leaf area (Neilson et al., 2015). The produced leaf area during vegetative stages is closely associated with the growth rate at flowering, which in turn increases the seed number (Moser et al., 2006; Borras et al., 2009). Both the plant size and leaf area, among other traits, are reported to contribute as constitutive traits in dehydration avoidance (ability to sustain tissue hydration under drought) in drought-resistant cereal cultivars (Blum, 2005).

A faster decrease of correlation coefficients between the EBv and other image-derived traits with increasing stress intensity suggests that different physiological mechanisms and genes may be involved in adaptation for higher stress (Banziger and Edmeades, 1997). Although morphological image-derived traits remain highly correlated with the EBv also in stress conditions, color-related traits such as color ratios (Y2G and brown to green) showed a more durable significant effect on the EBv than morphological traits (except CLe). Color ratios may help to determine a different stress status and its effects on the phenotype (Chen et al., 2014), and since they are highly heritable (in the range of 0.60 and above), they can be used in studies to investigate the genetics of wilting processes (Neumann et al., 2015; Dodig et al., 2019). However, selecting not only the most appropriate traits but also the critical time for their evaluation is also essential to be determined (Araus and Cairns, 2014). The highest correlation between the secondary trait and the target trait (biomass or yield) is considered to be a critical stage from a breeding point of view. For example, few traits such as Lab_b, FI, and relative growth rate had exerted a particularly strong and timely effect on the EBv at the time of maximum water stress. Few traits such as solidity, SCov, and Lab_a had two periods of significant effects on the EBv: first, at earlier growth stages when no or little stress was present (up to DAT 12–13) and again at the maximum water stress (DAT 22–23). Only FI can be used to differentiate stressed genotypes after maximum stress (DAT 23–29). This is in agreement with the statement that a single chlorophyll fluorescence level can be used to distinguish between non-stressed and senescent area primarily at later stages of stress development (Humplík et al., 2015). The FI estimation based on color detection by RGB imaging allows the detection of senescence, necrosis, and chlorosis, but it is not suitable for measuring photosynthetic activity since the lighting system is not pulsed. In general, under no stress or mild stress conditions, morphological traits were more appropriate than color-related traits for the prediction of biomass accumulation. Nevertheless, under more severe stress conditions, color-related traits and FI would be more useful to differentiate genotypes for high biomass. Both types of traits (morphological and physiological) are shown to be more indicative for biomass accumulation earlier than later in the season. This may imply that the number and the size of genetic factors operative at later stages are not much larger and much higher in effect than those acting at early stages (Muraya et al., 2017).

To analyze whether ILs can express the EBv in similar patterns, temporal profiles were clustered by using the fuzzy c-means clustering method. Typical temporal profiles could provide better distinction of genetic differences in the time dimension (Han et al., 2019a). We have identified two temporal dynamics of EBv patterns among the studied ILs in each treatment (named groups A and B). Basically, in each treatment, groups were distinguished by the size of the EBv. Group A had a constantly higher EBv and plant size (i.e., PHg and CLe) through the experiment than group B. However, the difference was bigger at the beginning of the experiment than in the end due to a constantly higher relative growth rate of group B than group A by 14, 16, 20, and 19% in C, N, W, and NW, respectively. Furthermore, group B showed a strong increase in solidity and SCov (proxies for the leaf area index) compared with group A, being lower at the beginning but increasing until the end of the experiment. The leaf area index is defined as one half the total/green leaf area per unit horizontal ground surface and plays an important role in processes such as canopy interception, evapotranspiration, and gross photosynthesis (Yang et al., 2009). A recent study showed leaf area index dynamics as a promising trait in crop breeding (Blancon et al., 2019).

Color ratios at maximum stress (at DAT 22 in W and NW; at the end of experiment in N) were lower in group B, suggesting a lower level of stress in group B compared with group A. Indeed, group B had better DTO than group A. The typical growth curve of groups A and B for the period between the maximum growth before maximum stress and the day following maximum stress (DAT 19–23) showed that the EBv in group B started to decrease 1 day later than in group A. Moreover, a relative decline of the EBv immediately following the maximum growth before DAT 22 was lower in group B than in group A for 4–5%. It seems that smaller plants (group B) faced less severe drought than taller plants (group A). Different genotypes might have experienced different stress levels due to differences in biomass. A smaller plant/genotype would have a lower demand for water than a taller one. Indeed, SFC at maximum stress (DAT 22) was significantly different among ILs in both water stress treatments, ranging from 19.3 to 24.3% (W) and from 19.5 to 24.7% (NW). These differences were in significant negative correlations with maximum EBv growth before maximum water stress in W (*r* = −0.605^∗∗^) and NW (*r* = −0.502^∗^). Both parameters, the EBv decline in days and its relative decrease following maximum growth, may characterize timing and quantity of wilting symptoms in each IL, respectively. In this regard, IL 16 (1 day of decline and 1% of decrease), IL 3 (1 day and 3%), and IL 17 (1 day and 4%) were the most drought-resistant ILs in both water stress treatments. In addition, these ILs were among the top ranked for DTO based on the relative growth calculated for the period between water stress initiation and maximum water stress. The genetic gain in maize yield is not associated with yield potential *per se*, or with heterosis *per se*, but rather with increased stress tolerance, and the potential for the future yield improvement through increased stress tolerance of maize is still large (Tollenaar and Lee, 2002; Duvick, 2005). Interestingly, the improvement of mid-season DTO in maize resulted also in improved performance under low nitrogen stress, without significant yield penalties under optimal input conditions (Banziger et al., 1999; Zaidi et al., 2004). Indeed, our previous study (Dodig et al., 2019) showed that high resilient/tolerant and high productive ILs had significantly better physiological nitrogen use efficiency than low resilient/tolerant and low productive ILs under nitrogen stress treatment. Interestingly, when we compared fuzzy c-mean cluster group A and group B for mean physiological nitrogen use efficiency, there was no difference between them under nitrogen stress. However, under W and NW, group B showed better physiological nitrogen use efficiency than group A, suggesting that better nitrogen use under water stress may help DTO. This needs further investigation given the fundamental importance of water and nitrogen supply to the success of sustainable crop production.

Drought recovery is defined as the capability of plants to resume growth and to yield after drought events that altered plant water relations (Luo, 2010; Fang and Xiong, 2015). While group B had better DTO than group A, the latter had better DRC and DAD. The strongest DRC in W and NW was recorded in ILs 6, 9, 12, 13, and 19, all belonging to group A. The correlation analysis confirmed a weak relationship between DTO and DRC. DTO was in positive and significant correlation with RGR and WUE, but not with plant size (maximum EBv before wilting point) in both water-limiting settings. On the other side, DRC was in positive correlation with WUE but not with RGR. Although plant size had a positive effect on DRC, it was significant only in W. This is in agreement with previous studies, which reported that drought and subsequent recovery had been independently regulated processes (Chen et al., 2016; Lyon et al., 2016). DTO and DRC are key determinants of plant drought adaptation (Chen et al., 2016). Our results suggest that DRC may play a more critical role than DTO in DAD. This is partly in agreement with Chen et al. (2016) who reported that DAD is closely related to DRC but not to DTO. Among the five ILs with the highest DAD in W (ILs 6, 8, 12, 16, and 19), IL 8 and IL 16 had high DTO, while IL 6, IL 12, and IL 19 had high DRC. Several ILs had high tolerance but low recovery (e.g., ILs 3, 7, and 17) and *vice versa* (ILs 9 and 13). The same is in NW. The best performing ILs for drought-adaptive capabilities were IL 6 (high DRC and DAD, and average DTO) and IL 8 (high DTO and DAD, and average DRC). ILs 6 and 8 belong to different groups, A and B, respectively, in both water stress treatments. This suggests that the ILs with different temporal profiles had different strategies to cope with drought stress. Avramova et al. (2016) showed that drought-tolerant maize hybrids of European and African origin differed in acclimation of the shoot and root growth and development in response to drought stress. Thus, these two ILs with different growth trajectories in our study could theoretically be crossed to pyramid a new genotype with a desirable growth phenotype under water stress. Finally, the obtained results on drought capabilities fit well to the initial classification of susceptible (ILs 1–5) or tolerant (ILs 6–20) as known from previous field experiments (Supplementary Table 1). However, future work will consider releasing a dataset from field trials in order to make research more broadly relevant.

Finally, to define the roles of morphological and physiological traits in plant drought adaptation, a correlation analysis between drought-adaptive capabilities and image-derived traits was conducted at two time points. A lower number of significant correlations found at DAT 35 compared with DAT 22 indicated that drought stress-related traits should be studied during or immediately after the application of maximum stress. Under maximum water stress, accumulated biomass, relative growth rate, and proxies for the leaf area index (solidity and soil coverage) can be considered, among the studied traits, as the most relevant indicators of DTO and DAD. Furthermore, lower color ratios (i.e., more green biomass) will contribute to better DTO. More greenness means less damaged tissue, which may contribute to the better maintenance of photosynthesis. Both biomass-related traits (aerial biomass and relative growth rate) and leaf area are basic growth traits proved to be the key indicators related to water deficit in maize (Sinclair et al., 1990; Tollenaar and Lee, 2006; Cairns et al., 2012; Chapuis et al., 2012; Paredes et al., 2014). For example, the results of the study of Chapuis et al. (2012) indicated that the capacity to maintain leaf growth under water deficit in controlled conditions was consistent with high grain yield in different field drought environments. Several other non-morphological and non-color-related traits were reported to contribute to DTO such as leaf water potential, chlorophyll content, starch content, total non-structure carbohydrate, nitrogen content, and Fv/Fm (Chen et al., 2016). Inbred lines with higher accumulated biomass at DAT 35 had better DRC and DAD, but not tolerance. However, DTO can be improved by relative growth rate. Interestingly, only the EBv and plant compactness (only in NW) showed a significant relationship with DRC at DAT 35, but not at DAT 22, suggesting different physiological bases of DTO and DRC. It seems that genetically determined differences in the DRC were too small to be detected by morphological and color-related parameters used in this study. Recovery after stress in maize involved multiple physiological and metabolic processes to repair drought-induced injuries and resume plant growth (Avramova et al., 2015; Chen et al., 2016; Zhang et al., 2018). The studies of Chen et al. (2016) and Zhang et al. (2018), for example, showed that reducing the damage caused by drought on the plant photosynthetic system and the chlorophyll content contributes to a better recovery after rewatering. Obviously, a set of only six morphological and five physiological (mainly color-related) traits was not sufficiently large to capture these complex biological processes related to recovery. However, the latter upgraded the system at IPK, which now supports the assessment of approximately 200 phenotypic traits, including kinetic chlorophyll fluorescence (Tschiersch et al., 2017), which may serve as a useful resource for future investigations of drought-responsive traits.

## Conclusion

In this study, we have used non-invasive and high-throughput phenotyping for the quantitative assessment of dynamic growth and architectural patterns during drought across vegetative maize development. The accumulated biomass, relative growth rate, leaf area index, and color ratios under water stress were identified as drought- and recovery-responsive traits and could be used as reference indicators in the selection of drought-adaptive genotypes. The relative growth rate, leaf area index, and greenness of the plant were related to DTO, whereas the EBv was indicative of DRC. The correlation analysis suggests that DRC may play an equal role as DTO in DAD. In general, under no stress or mild stress conditions, morphological traits were more appropriate than color-related traits for predicting biomass accumulation. Nevertheless, under more severe stress conditions, color-related traits and FI would be more useful to differentiate the genotypes’ high biomass production ability. Genotypes with high DAD but different growth trajectories could be theoretically crossed to pyramid a new genotype with a desirable growth phenotype under water stress.

## Data Availability Statement

The raw data supporting the conclusions of this article will be made available by the authors, without undue reservation.

## Author Contributions

DD, SB, JV, DI-M, and ND conceived and designed the research. SB, AN, and KW-F performed the experiment. MZ, SB, and DD analyzed and interpret the data. DD wrote the original manuscript with input from SB and MZ. TA and AJ supervised the project. AJ critically revised the manuscript. All authors contributed to the article and approved the submitted version.

## Conflict of Interest

The authors declare that the research was conducted in the absence of any commercial or financial relationships that could be construed as a potential conflict of interest.
